# Structural Diversity and Biological Activities of Novel Secondary Metabolites from Endophytes

**DOI:** 10.3390/molecules23030646

**Published:** 2018-03-13

**Authors:** Han Gao, Gang Li, Hong-Xiang Lou

**Affiliations:** 1 Department of Natural Medicine and Pharmacognosy, School of Pharmacy, Qingdao University, Qingdao 266021, China; hg201602@163.com (H.G.); gang.li@qdu.edu.cn (G.L.); 2 Department of Natural Product Chemistry, Key Laboratory of Chemical Biology of Ministry of Education, School of Pharmaceutical Sciences, Shandong University, No. 44 West Wenhua Road, Jinan 250012, China

**Keywords:** endophytes, natural products, skeletons, biosynthesis, bioactivities

## Abstract

Exploration of structurally novel natural products greatly facilitates the discovery of biologically active pharmacophores that are biologically validated starting points for the development of new drugs. Endophytes that colonize the internal tissues of plant species, have been proven to produce a large number of structurally diverse secondary metabolites. These molecules exhibit remarkable biological activities, including antimicrobial, anticancer, anti-inflammatory and antiviral properties, to name but a few. This review surveys the structurally diverse natural products with new carbon skeletons, unusual ring systems, or rare structural moieties that have been isolated from endophytes between 1996 and 2016. It covers their structures and bioactivities. Biosynthesis and/or total syntheses of some important compounds are also highlighted. Some novel secondary metabolites with marked biological activities might deserve more attention from chemists and biologists in further studies.

## 1. Introduction

The last 30 years have seen tremendous successes in natural product-based drug discovery [1,2]. Natural products, their semisynthetic derivatives, and synthetic products that mimic a natural product template, represent more than half of all approved small-molecule drugs [1,3]. Diverse and biologically active pharmacophores, especially in naturally occurring novel compounds, play a pivotal role in modern drug discovery [4,5]. They possess specific steric and electronic properties for molecular recognition by a biological target [6]. Alarmingly, only a few new natural product drug pharmacophores have been discovered in the last twenty years, which poses critical issues for natural product-driven lead discovery campaigns and new drug types [7].

Many strategies have been developed to discover structurally novel natural product leads through available biological approaches [8]. Mining the largely unexplored natural sources, such as endophytes, will pave the way for chemical and biological novelties [8]. Endophytes, mainly fungi and bacteria, colonize the living, internal plant tissues without causing visible symptoms of disease [9]. There are approximately 300,000 different plant species inhabiting our planet and it can be expected that each individual one has a complex community of one to many cultivable or uncultivable endophytic microorganisms [10,11]. Endophytes are recognized to have complex associations with host plants and other organisms, including endophytic microorganisms in their ecological niches and pathogens in external environments [12,13,14]. In order to adapt to their microenvironments, endophytes typically coevolve a plethora of traits that range from production of diverse chemical defense compounds to triggers for activating cryptic biosynthetic pathways, production of precursors, quorum sensing molecules, epigenetic modulators, and even direct physical organismal interactions [15,16]. These functional biomolecules derived from endophytes are important from an ecological perspective [13]. For instance, the endophytic fungus *Neotyphodium coenophialum* inhabiting the tall fescue (*Festuca arundinacea*) was discovered to produce toxic alkaloids, defending host plants against herbivorous mammals and causing “fescue toxicosis” of livestock [17]. From the medicinal perspective, they may directly or indirectly be used as therapeutic agents against numerous diseases.

The enormous diversity of endophytes in combination with their potential biosynthetic capabilities has provided the impetus for a number of chemical investigations on endophytes. Endophytes are now well-known to biosynthesize diverse natural products with intriguing biological activities, and around ten reviews have reported on the new and known bioactive secondary metabolites of endophytes [18,19,20,21,22,23,24,25,26,27]. It should be noted that small molecules with new carbon skeletons, unusual ring systems, or rare structural moieties from endophytic fungi and bacteria have not been reviewed to the best of our knowledge. They might deserve attention from chemists and biologists and could be a potential resource of new biologically active pharmacophores for natural product-based drug development.

The target of this review is to summarize endophyte-derived secondary metabolites with new carbon skeletons, unique ring systems, or uncommon structural moieties isolated in a period between 1996 and 2016 that marks enormous progress in the chemical investigation of fungal and bacterial endophytes. Their structures and biological activities, together with the biosynthesis and total syntheses of some important molecules are described. In this review, the structures are mainly classified according to their proposed biosynthesis. They might be further arranged according to the structural features of secondary metabolites.

## 2. Polyketides

### 2.1. Macrolides

**Figure 1:**
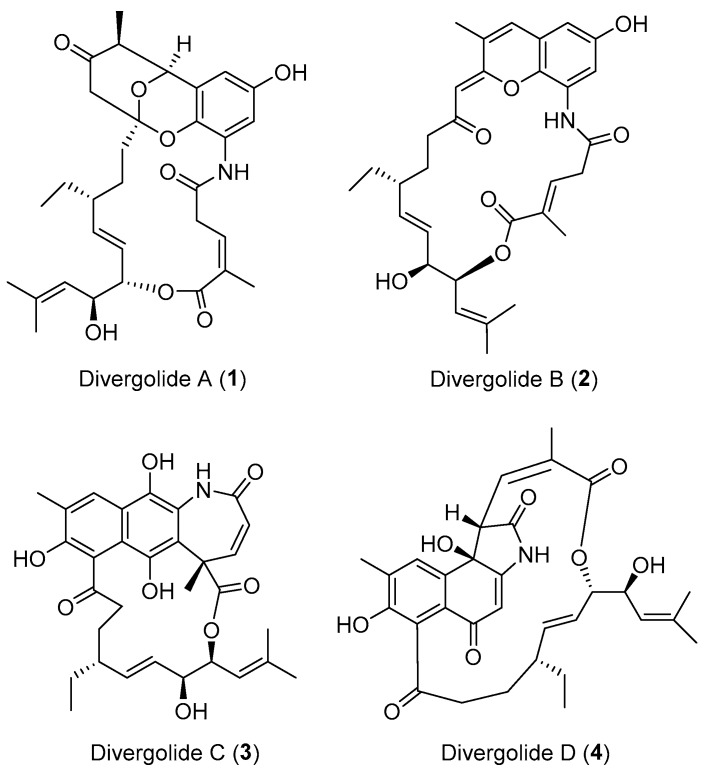
Structures of divergolides A–D (**1**–**4**).

A mangrove-derived bacterial endophyte *Streptomyces* sp. was discovered by the Hertweck group to produce four unprecedented ansa macrolides, divergolides A–D (**1**–**4**, Figure 1) [28,29].

**Scheme 1:**
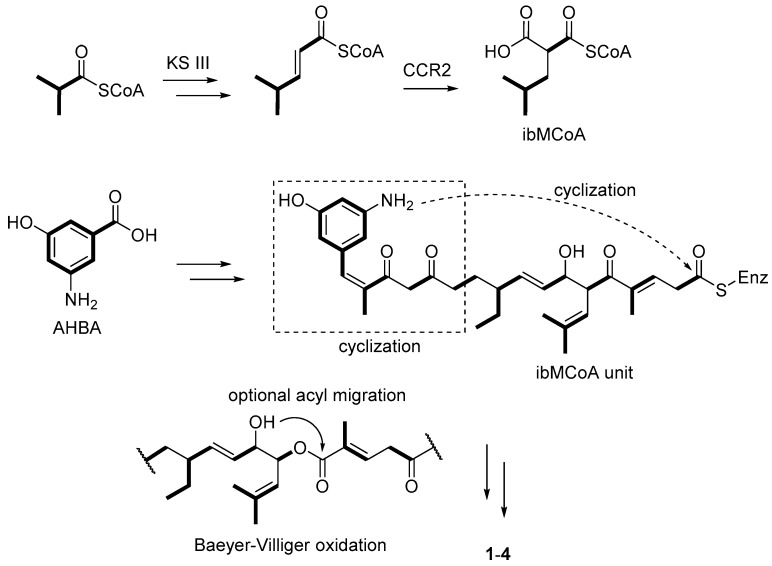
The proposed biosynthetic pathway for divergolides A–D (**1**–**4**), starting from the ansamycin starter unit AHBA. The polyketide backbone is proposed to be disrupted through a putative Baeyer-Villiger oxidation.

They were biosynthesized from a common linear polyketide precursor that underwent various reactions including an optional acyl migration to form the diverse multicyclic structures (Scheme 1). An unusual isobutylmalonyl-CoA (ibMCoA) extender unit derived from isobutyrate and acetate rather than l-leucine was involved in the divergolide polyketide pathway (Scheme 1) [30]. The remarkable structural plasticity of this kind of macrolides led to different antibacterial and cytotoxic properties [31]. Compound **1** showed the strongest antibacterial activity against *Mycobacterium vaccae* with an inhibitory zone of 19 mm at 50 μg per paper disk in the disk diffusion assay, while compound **4** demonstrated marked cytotoxicity against several cancer cell lines, with IC_50_ values ranging from 1.0 to 2.0 μM [28]. Their intriguing structures and associated antibacterial or antitumor activities have stimulated various synthetic methods towards divergolides [32,33], and scientific interest in biosynthetic gene clusters [29].

**Figure 2:**
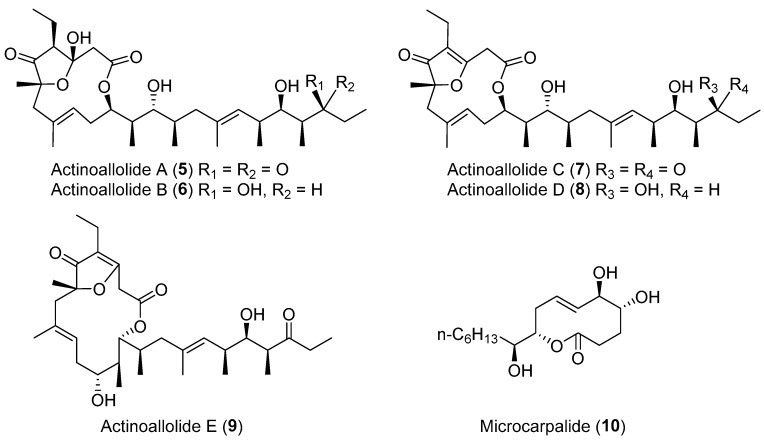
Structures of actinoallolides A–E (**5**–**9**) and microcarpalide (**10**).

Iwatsuki and co-workers obtained a fungus *Actinoallomurus fulvus* harbored in the roots of *Capsicum frutescens* collected in Thailand [34]. Chemical investigation of this fungus led to the discovery of five unique 12-membered macrolides, actinoallolides A–E (**5**–**9**, Figure 2). Compound **5** exhibited significant anti-trypanosomal activity against *Trypansoma cruzi* (IC_50_: 0.226 μg/mL) similar to that of commonly used therapeutic drug, benznidazole (IC_50_: 0.418 μg/mL), indicating a promising new class of lead compounds for treating Chagas disease [34]. Bioassay-guided isolation of the ethyl acetate extract of an unidentified endophytic fungus provided an unusual C_16_ nonenolide, microcarpalide (**10**) (Figure 2) with an alky side chain. Compound **10** disrupted microfilaments in approximately half of the cells at a concentration of 0.5–1.0 μg/mL and showed weak cytotoxicity against two mammalian cell lines (KB and LoVo) [35].

### 2.2. Benzopyran

**Figure 3:**
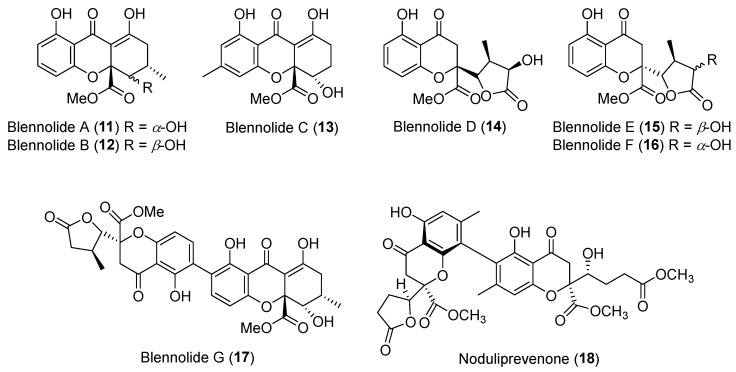
Structures of blennolides A–G (**11**–**17**) and noduliprevenone (**18**).

The Krohn group discovered seven rare chromanones, blennolides A–G (**11**–**17**, Figure 3) from an endophytic fungus *Blennoria* sp. occurring in *Carpobrotus edulis* found in the Canary Islands [36]. They displayed moderate antialgal activity against *Chlorella fusca* and antifungal activity against *Microbotryum violaceum* with radii of the zones of inhibition ranging from 5 to 9 mm with 50 μg per paper disk in the agar diffusion assay. Compounds **14**–**16** are unique natural products with a highly substituted *γ*-lactone moiety, while compound **17** is a novel heterodimer incorporating two unusual chromanone subunits, the monomer **11** and the deoxy analogue of monomer **15** [36]. Another unusual heterodimeric chromanone, noduliprevenone (**18**) (Figure 3), was isolated from a Mediterranean alga-derived endophyte *Nodulisporium* sp., and was a potential competitive inhibitor of cytochrome P450 1A with an IC_50_ value 6.5 ± 1.6 μM [37].

**Figure 4:**
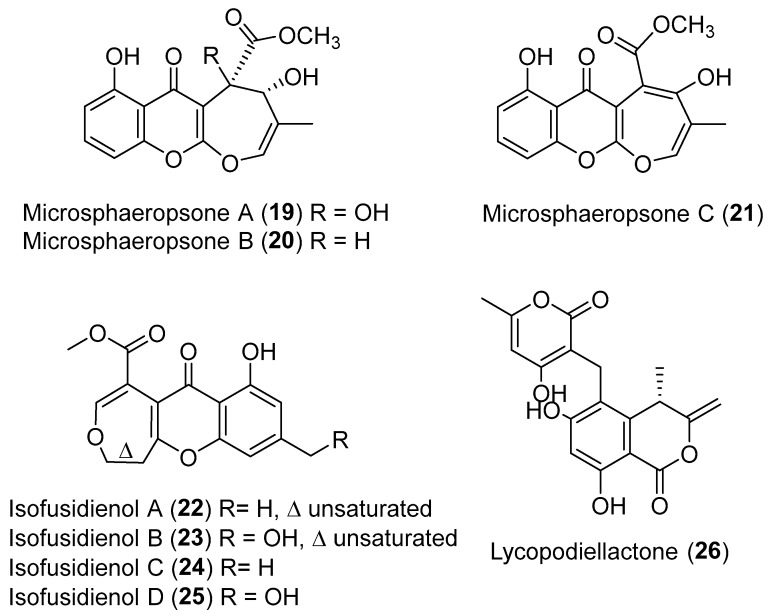
Structures of microsphaeropsones A–C (**19**–**21**), isofusidienols A–D (**22**–**25**) and lycopodiellactone (**26**).

In the course of discovering novel and bioactive metabolites from endophytic fungi, the Krohn group found three more novel antimicrobial benzopyran derivatives, microsphaeropsones A–C (**19**–**21**, Figure 4) with a unique oxepino[2,3-*b*] chromen-6-one (ring-enlarged xanthone) skeleton from an endophyte *Microsphaeropsis* sp. isolated from the shoots of *Lycium intricatum* [38]. From an endophytic *Chalara* sp. isolated from the plant *Artemisia vulgaris*, isofusidienols A–D (**22**–**25**) (Figure 4) with an unprecedented chromone-3-oxepine moiety were found by the Zeeck group. Compounds **22** and **23** exhibited strong antibacterial activity against *Bacillus subtilis* with inhibition zones of 23 and 22 mm at 15 μg/disk, respectively [39]. Lycopodiellactone (**26**, Figure 4) with an uncommon *δ*-lactone and a rare 3-methylene isochromanone moiety, was obtained from a fungal endophyte *Paraphaeosphaeria neglecta* isolated from a Hawaiian indigenous plant, *Lycopodiella cernua* [40]. This metabolite might be biosynthesized by a polyketide pathway involving a key condensation of the *δ*-lactone and the 3-methylene isochromanone motif.

### 2.3. Spiro Compounds

**Figure 5:**
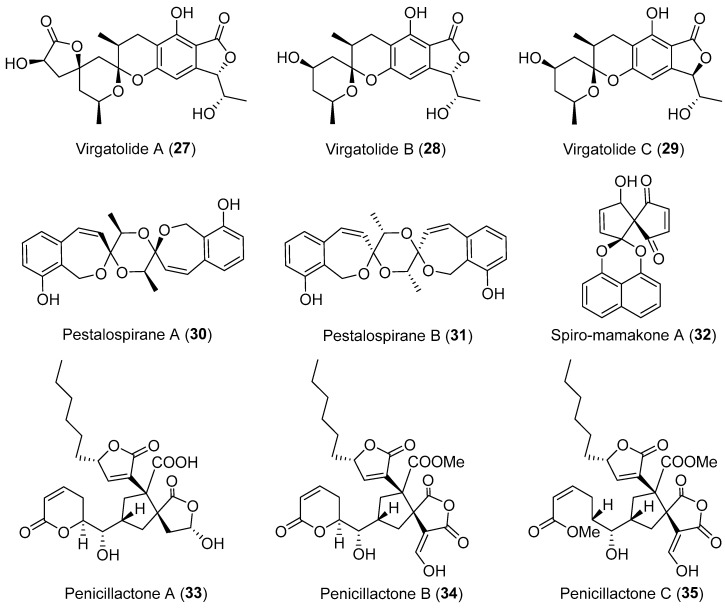
Structures of compounds **27**–**35**.

**Scheme 2:**
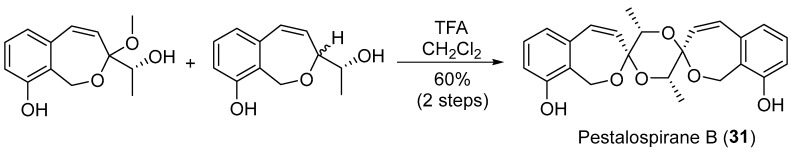
Bioinspired tandem dimerization-spiroketalization for the total synthesis of pestalospirane B (**31**).

Chemical investigation of the EtOAc extract of an endophytic fungus *Pestalotiopsis virgatula* led to the isolation of three cytotoxic metabolites named virgatolides A–C (**27**–**29**, Figure 5) [41]. They are new members of the rare benzannulated 6,6-spiroketal class of natural products and possess one or two *γ*-lactone units, representing the first occurrence of the *γ*-lactone units in the benzannulated 6,6-spiroketals. Jaroszewski and co-workers employed a hyphenated technique comprising HPLC-SPE-NMR to uncover some novel metabolites from *Pestalotiopsis virgatula*, an endophyte inhabiting the bark of *Terminalia chebula* [42,43]. Among them, pestalospiranes A and B (**30** and **31**, Figure 5) have an unprecedented 1,9,11,18-tetraoxadispiro[6.2.6.2]octadecane skeleton in addition to the characteristic benzo[*c*]-oxepin motif [43,44]. A bioinspired tandem dimerization-spiroketalization strategy to forge the unique dispiro skeleton of **31** has recently been described (Scheme 2) [44].

The Munro group from New Zealand disclosed the structure of spiro-mamakone A (**32**, Figure 5) from a non-sporulating endophytic fungus derived from the New Zealand native tree *Knightia excels* [45]. This compound belongs to the family of the structurally diverse spirobisnaphthalenes and represents the first spirobisnaphthalene analogue containing a new spiro-nonadiene skeleton [45]. Using feeding experiments conducted with different labeled acetates, the biosynthesis of compound **32** was investigated and found to involve the same two pentaketide-derived naphthalene units that underwent oxidative coupling and further extensive rearrangement [46]. Compound **32** exhibited significant cytotoxicity toward the P388 murine leukemia cell line (IC_50_ of 0.33 μM), and was also active against three selected bacteria [45]. A series of spiro-mamakone analogues have been synthesized for the investigation of structure-activity relationships, confirming the importance of the enedione moiety to bioactivities [47]. Penicillactones A–C (**33**–**35**, Figure 5) were biosynthesized by an endophytic fungus, *Penicillium dangeardii* residing in the plant *Lysidice rhodostegia*, and are novel natural products possessing a spirocyclic anhydride moiety [48]. Compounds **34** and **35** were active in inhibiting the release of *β*-glucuronidase from polymorphonuclear leukocytes with ED_50_ values of 2.58 and 1.57 μM, respectively.

### 2.4. Quinones

**Figure 6:**
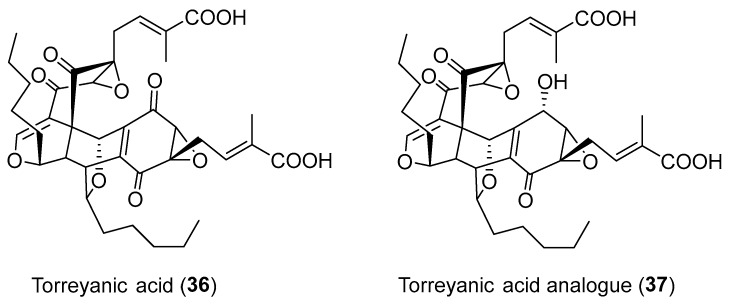
Structures of (±)-torreyanic acid (**36**) and its analogue **37**.

In 1995, Clardy and co-workers identified an endophytic fungus *Pestalotiopsis microspora*, which lived in the inner bark of the healthy host plant *Torreya taxifolia* but could be switched to have the pathological activity by environmental triggers [49]. Bioassay-guided investigation of the fermentation culture of *P. microspora* led to the isolation of an unusual dimeric quinone, (±)-torreyanic acid (**36**, Figure 6) [50]. Compound **36**, as a cytotoxic agent, caused cell death by apoptosis with IC_50_ values ranging from 5.1 to 65.0 μM for 25 different human cell lines [50]. Inspired by a proposed biosynthetic scheme of **36**, the Porco, Jr. and Mehta groups successfully applied a biomimetic electrocyclization/Diels-Alder dimerization cascade to construct the structure of **36** [51,52]. Ding et al. isolated and identified a novel torreyanic acid analogue (**37**, Figure 6) from a fungus *Pestalotiopsis* sp. inhabiting the lichen *Clavaroids* sp. [53].

### 2.5. Nitrogen-Containing Heterocycles

**Figure 7:**
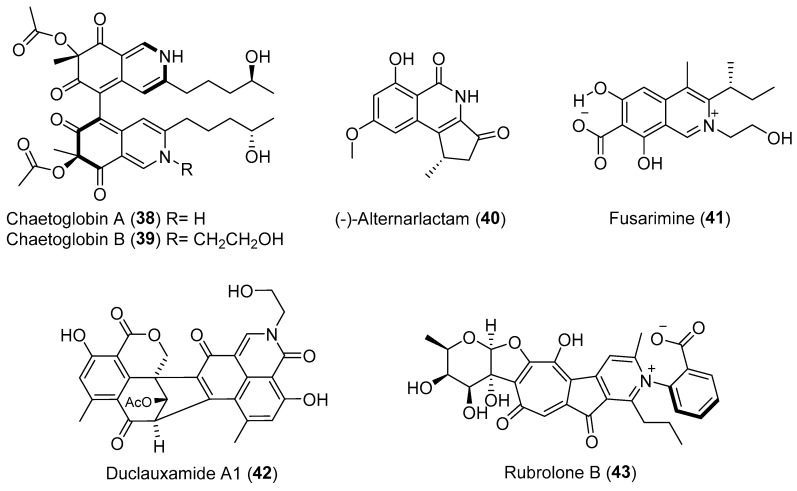
Structures of compounds **38**–**43**.

Chaetoglobins A (**38**) and B (**39**) (Figure 7), the first azaphilone alkaloid dimers formed through bonding between C-5 and C-5′ [54], were isolated from a fungus, *Chaetomium globosum,* residing inside the stem of *Imperata cylindrical* by the Tan group [55,56]. Compound **38** has been demonstrated to be significantly cytotoxic against the human breast cancer cell line MCF-7 and colon cancer cell line SW1116 with IC_50_ values of 42.1 and 55.7 μM, respectively. (−)-Alternarlactam (**40**, Figure 7), as a unique polyketide, was also described firstly by the Tan group and was obtained from a strain of *Alternaria* living inside the leaves of *Carex aridula* [57]. Compound **40** contains two important antitumor-related pharmacophores, cyclopentenone and isoquinolinone scaffolds, and was highly effective against human cervix HeLa adenocarcinoma cell and human hepatocellular carcinoma cell with IC_50_ of 4.2 μM and 5.9 μM, respectively. The total synthesis of **40** has been achieved through using two commercially available chemicals, 3,5-dimethoxyaniline and (±)-4-methyl-1,2-cyclo-pentanedione [57].

**Scheme 3:**
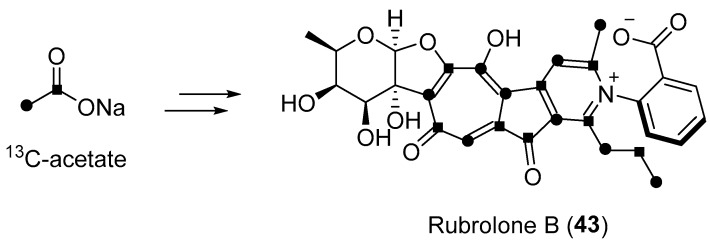
Biosynthesis of rubrolone B (**43**) on the basis of the data from feeding experiments.

The Gao group reported a polyketide-derived isoquinoline alkaloid, fusarimine (**41**, Figure 7) containing a rare *N*-ethyl-4-methyl-7-carboxyisoquinoline carbon skeleton [58]. This compound can be derived biogenetically from a single hexaketide chain with an external nitrogen incorporated in the endophytic fungus *Fusarium* sp. occurring in the renowned insecticidal plant *Melia azedarach*. Duclauxamide A1 (**42**, Figure 7) was purified from the endophytic *Penicillium manginii* inhabiting the elder root of the traditional Chinese medicinal (TCM) plant *Panax notoginseng* by the Huang group [59]. As a polyketide-derived heptacyclic oligophenalenone dimer with an uncommon *N*-2-hydroxyethyl moiety [60], compound **42** demonstrated moderate cytotoxicity against HL-60, SMML-7721, A-549, MCF-7, and SW480 cancer cell lines with IC_50_ values ranging from 11 to 32 μM. From another TCM plant *Camellia sinensis* selected by Huang and co-workers, a bacterial endophyte, *Streptomyces* sp. was isolated and was found to produce a purple red solid, rubrolone B (**43**, Figure 7) with potential cardioprotection [61]. This metabolite belongs to the tropolone alkaloid family [62,63], but displays an expanded aromatic tropolone skeleton that includes a unique benzoic acid-pyridine inner salt fragment. Feeding experiments using ^13^C-labled acetates indicated a type-II polyketide synthase (PKS)-catalyzed biosynthesis route followed by complex oxidative rearrangements to form the tropolone ring system (Scheme 3) [61].

### 2.6. Others

**Figure 8:**
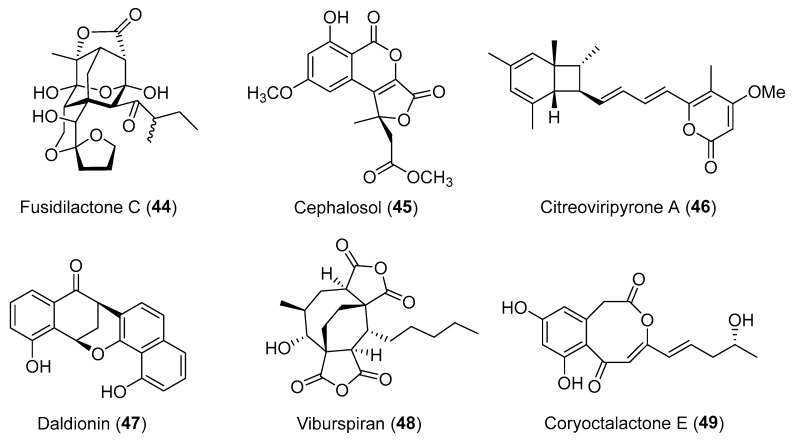
Structures of compounds **44**–**49**.

**Scheme 4:**
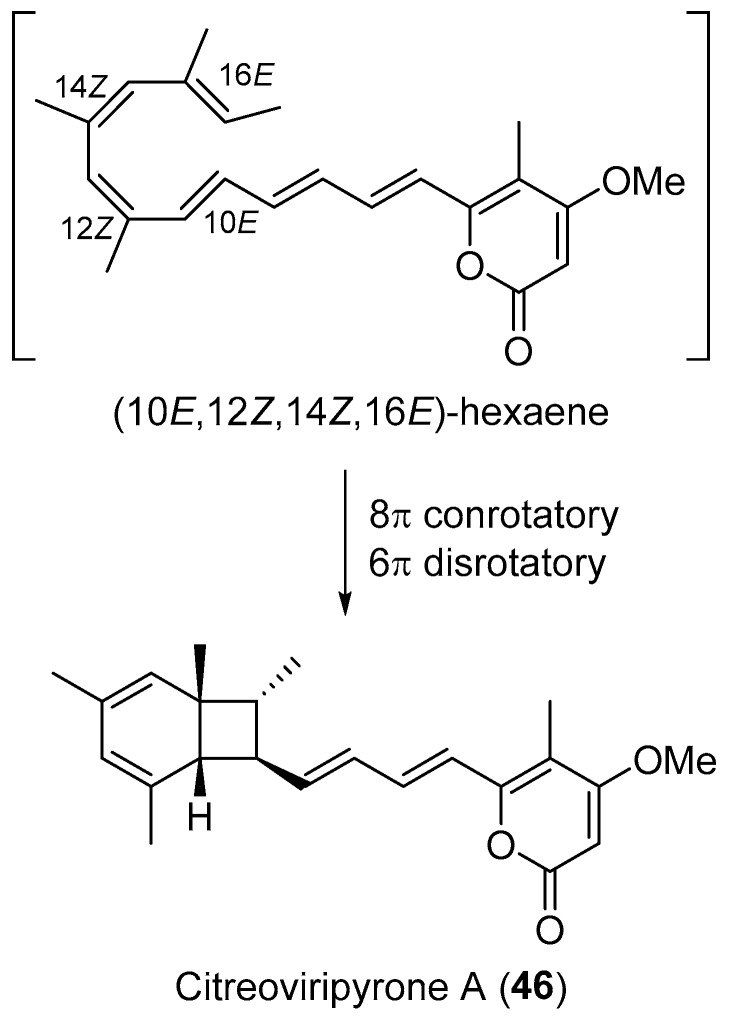
Plausible biosynthetic pathway of citreoviripyrone A (**46**).

Following an antimicrobial screening for bioactive metabolites from endophytic fungi [64], fusidilactone C (**44**, Figure 8) was purified and found to comprise an unusual and rigid oxoadamantane skeleton and also has two ether-bridged hemiacetals in addition to its spiro acetal structure [65,66]. A novel ketal-tethered intramolecular Diels-Alder cycloaddition has been developed for the synthesis of the 2-oxadecalin spiroketal core of **44** [67]. Cephalosol (**45**, Figure 8), isolated from *Cephalosporium acremonium* that used to reside as an endophyte in *Trachelospermum jasminoides*, showed strong antimicrobial activities against *Escherichia coli*, *Pseudomonas fluorescens*, *Trichophyton rubrum*, and *Candida albicans* with MIC values of 3.9, 3.9, 7.8 and 1.95 μg/mL, respectively [68]. Compound **45**, with an unprecedented carbon skeleton, was proposed to be derived from a PKS pathway similar to that of alternariol and graphislactones [69], and has already been a total synthesis target [70]. An endophytic fungus from the leaves of *Catharanthus roseus* was identified as *Penicillium* sp. by the Asai group [71]. It produced citreoviripyrone A (**46**, Figure 8) with a bicyclo[4.2.0]octadiene arising from a key 8π-6π electrocyclization cascade route (Scheme 4) [72]. Compound **46** was toxic to human HCT 116 cells with a GI_50_ value of 10.4 μM. Recently, Hertweck and co-workers reported a polyketide-derived antibiotic, daldionin (**47**, Figure 8) with an unprecedented oxane-linked binaphthyl ring system, obtained from an orchid endophyte [73]. Another endophytic fungus *Cryptosporiopsis* sp. isolated from tissues of *Viburnum tinus* proved to produce viburspiran (**48**, Figure 8) [74]. It was the first eight-membered maleic anhydride natural product with potential antifungal activity against *Microbotryum violaceum* and *Botrytis cinerea* with radii of inhibition zones of 6 and 10 mm at 50 μg per paper disk, respectively [74]. Chemical investigation of the EtOAc extract of the mangrove-derived endophyte *Corynespora cassiicola* isolated from *Laguncularia racemosa*, provided five unusual octalactone derivatives, such as coryoctalactone E (**49**, Figure 8) [75].

**Figure 9:**
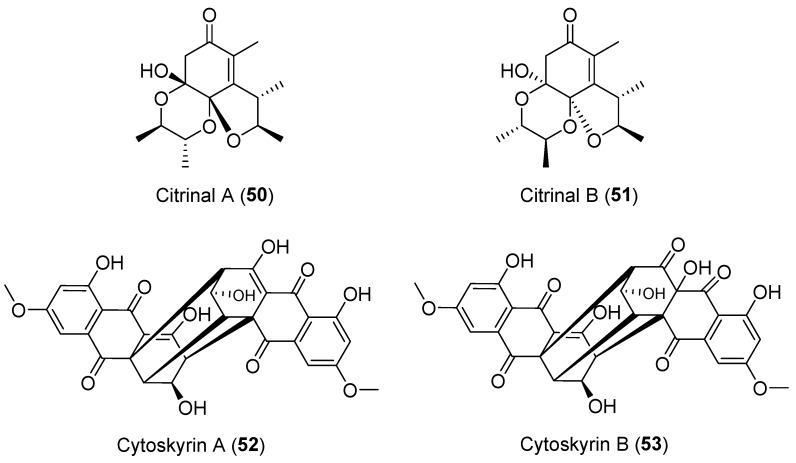
Structures of citrinals A–B (**50**–**51**) and cytoskyrins A–B (**52**–**53**).

Citrinals A and B (**50** and **51**, Figure 9) from the endophytic fungus *Colletotrichum capsici* represented a new compound class with a unique skeleton but displayed no cytotoxic activities [76,77]. Following a biochemical induction assay, cytoskyrins A and B (**52** and **53**, Figure 9) with a 1,3,6,8-tetrahydroxyanthraquinone-type carbon skeleton, were isolated from the endophytic fungus *Cytospora* sp. [78]. Compound **52** demonstrated strong biochemical induction assay (BIA; used to identify compounds that damage DNA or inhibit DNA synthesis) activity down to 12.5 ng while the biosynthetically related **53** was inactive. The total synthesis has already been reported by the group of Nicolaou by developing a cascade sequence called the “cytoskyrin cascade” [79,80,81].

## 3. Noribosomal Peptides

**Figure 10:**
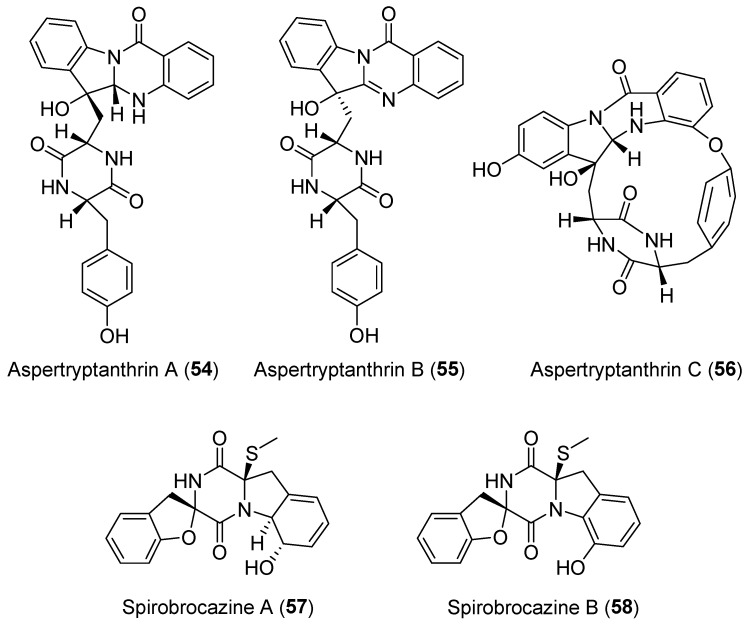
Structures of aspertryptanthrins A–C (**54**–**56**) and spirobrocazines A–B (**57**–**58**).

Aspertryptanthrins A–C (**54**–**56**, Figure 10), three new indole diketopiperazine alkaloids, were obtained from a strain of *Aspergillus* sp. isolated from the stem bark of *Melia azedarach* L. [82]. 

They possess a 6/5/6/6 tryptanthrin framework that is formed by a tryptophan unit and an anthranilate residue. In addition, compound **56** has an unusual 16-membered ring skeleton which was cyclized through the formation of phenylate. Spirobrocazines A and B (**57** and **58**, Figure 10) were isolated and identified from the mangrove-derived *Penicillium brocae* and possess a very rare spirocyclic skeleton [83]. Compound **57** showed moderate antibacterial activities against *E. coli*, *S. aureus*, and *Vibrio harveyi* with MIC values of 32.0, 16.0, and 64.0 μg/mL, respectively.

**Figure 11:**
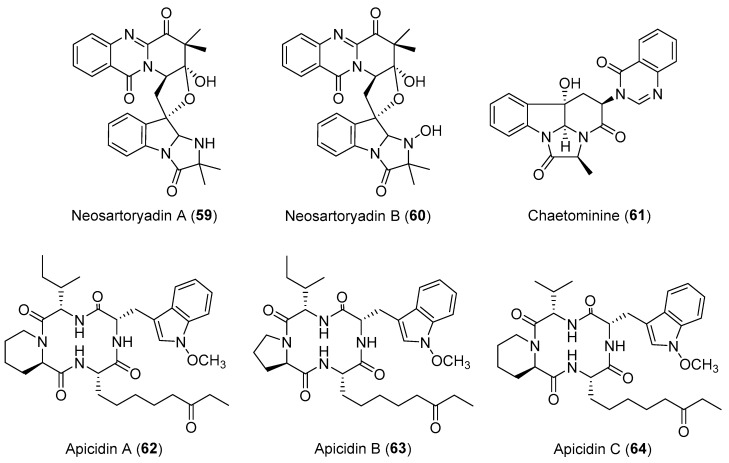
Structures of compounds **59**–**64**.

Neosartoryadins A (**59**) and B (**60**) (Figure 11) with a unique 6/6/6/5 quinazoline ring system connected directly to a 6/5/5 imidazoindolone ring system, represented a new class of quinazoline-containing indole alkaloids and displayed inhibitory effects against influenza A virus (H1N1) with IC_50_ values of 66 μM and 58 μM, respectively [84]. Their structures were proposed to be assembled by four amino acids l-tryptophan, anthranilic acid (ATA), l-valine, and 2-aminoisobutyric acid (Aib) in the endophytic fungus *Neosartorya udagawae* [84,85].

Antitumor screening of extracts of 43 endophytic fungi isolated from the leaves of the TCM plant *Adenophora axilliflora* enabled the discovery of a bioactive strain, *Chaetomium* sp. [86]. ^1^H-NMR and bioassay fractionation of the fungal culture led to the isolation of a tripeptide-derived alkaloidal metabolite, chaetominine (**61**, Figure 11) with a unique alanine-derived *δ*-lactam ring [87]. Compound **61** showed more potent cytotoxicity to the human leukemia K562 and colon cancer SW1116 cell lines than the positive drug 5-fluorouracil (IC_50_ values of 21.0 and 28.0 nM for compound **61**, respectively; IC_50_ values of 33.0 and 76.0 nM for 5-fluorouracil, respectively) [86]. It was proposed to be biosynthesized from l-alanine, ATA, and d-tryptophan and has been a target for numerous synthetic efforts [88]. Apicidins A–C (**62**–**64**, Figure 11), three new members of a unique family of cyclic tetrapeptides, were isolated from a fungal endophyte *Fusarium pallidoroseum* by chemists from Merck research laboratories [89,90]. They showed a variety of potent antiprotozoal activities by reversibly inhibiting histone deacetylase (HDAC) and are attracting considerable attention for their anti-tumor effects [91,92,93]. In particular, compound **64** showed MIC values of 0.8, 101, and 69 nM against *Besnoitia jellisoni*, *Eimeria tenella*, and *Plasmodium falciparum*, respectively, and was slightly more active than compounds **62** and **63** [90].

## 4. Isoprenoids

### 4.1. Steroids

**Figure 12:**
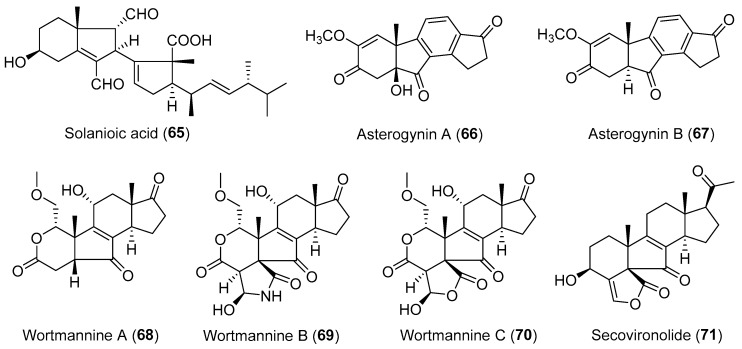
Structures of compounds **65**–**71**.

Solanioic acid (**65**, Figure 12), a degraded and rearranged steroid with an unprecedented carbon skeleton, has been isolated from the fungus *Rhizoctonia solani* obtained from tubers of the medicinal plant *Cyperus rotundus* [94]. It displayed significant inhibitory activities against *B. subtilis*, *S. aureus*, and methicillin-resistant *S. aureus* (MRSA) with MIC values around 1 μg/mL, and moderate antifungal activity against *C. albicans* with an MIC value of 16 μg/mL [94]. Asterogynins A (**66**) and B (**67**) (Figure 12), two unusual steroid-like metabolites with a tetracyclic carbocyclic ring system [95], were purified from the culture of *Chalara alabamensis* isolated from the host plant *Asterogyne martiana* [96]. More recently, four structurally related steroids, wortmannines A–C (**68**–**70**, Figure 12) and secovironolide (**71**, Figure 12) bearing an unusual five-membered B ring [97,98], were discovered from an endophytic fungus *Talaromyces wortmannii* living in *Tripterygium wilfordii* by the group of Yang.

### 4.2. Sesquiterpenoids

**Figure 13:**
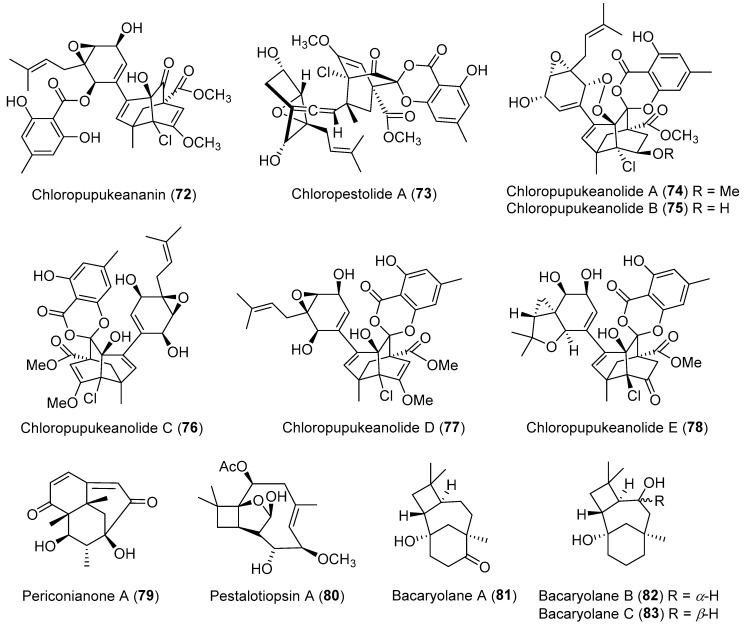
Structures of compounds **72**–**83**.

Chloropupukeananin (**72**, Figure 13) featuring a unique tricyclo-[4.3.1.03,7]-decane skeleton, was the first chlorinated pupukeananae derivative originated from a sesquiterpenoid in the plant endophyte *Pestalotiopsis fici* [99,100]. It inhibited the HIV-1 replication in C8166 cells at an IC_50_ of 14.6 μM and also exhibited weak antibacterial activity [100]. A key intermolecular Diels-Alder reaction followed by a subsequent carbonyl-ene reaction was proposed to be involved in the biosynthesis of compound **72** [100,101]. More novel pupukeananae derivatives with significant anti-HIV or anticancer activities, such as chloropestolide A (**73**) [102] and chloropupukeanolides A–E (**74**–**78**, Figure 13) [103,104], have also been reported from endophytes. Compounds **73** and **74** showed inhibitory effects on replication of the HIV-1 virus in C8166 cells with IC_50_ values of 62.4 and 6.9 μM, respectively, and inhibited the growth of HeLa cell line with IC_50_ values of 0.7 and 16.9 μM, respectively [102,103]. Compounds **76** and **77** demonstrated significant cytotoxicity against HeLa and HT29 cell lines with IC_50_ values ranging from 1.2 to 7.9 μM [104]. 

**Scheme 5:**
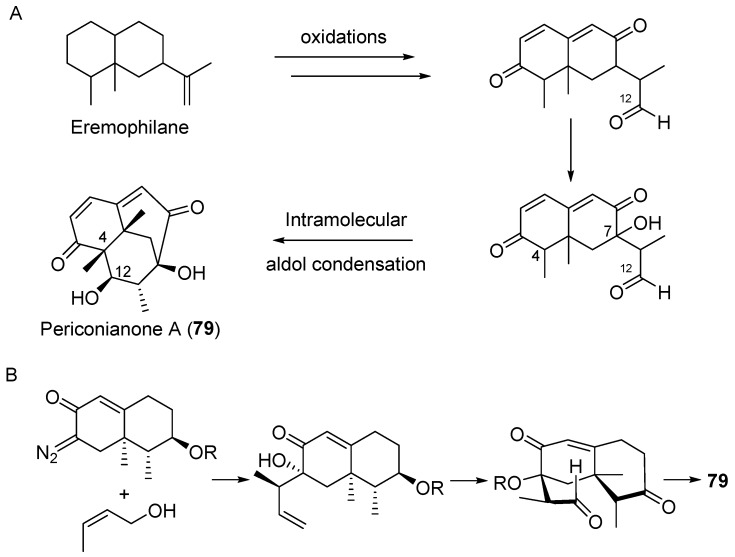
(**A**) Proposed biosynthetic pathway for periconianone A (**79**); (**B**) Synthetic strategy to **79** inspired by the biogenetic hypothesis of **79**.

Periconianone A (**79**, Figure 13), the first member of sesquiterpenoids with a new 6/6/6 tricarbocyclic skeleton, was isolated from *Periconia* sp. derived from the medicinal plant *Annonsa muricata* [105]. An intramolecular aldol condensation for the formation of a carbon bond between C-4 and C-12 might result in the generation of the unusual six-membered carbonic ring, which has recently been utilized in the totally synthetic strategy to **79** (Scheme 5) [106].

Compound **79** exhibited significant neural anti-inflammatory activity against lipopolysaccharide (LPS)-induced NO production in mouse microglia BV2 cells with IC_50_ value of 0.15 μM (curcumin as a positive control, IC_50_ = 3.9 μM) [105]. Pestalotiopsin A (**80**, Figure 13), an immunosuppressive agent, was isolated from an endophytic fungus Pestalotiopsis sp. associated with Taxus brevifolia by the group of Clardy [107]. The oxatricyclic ring system in the sesquiterpenoid **80** is unprecedented among natural products. In 2015, Ding et al. isolated three plant-like sesquiterpenes, bacaryolanes A–C (**81**–**83**, Figure 13) from a mangrove-derived bacterial endophyte Streptomyces sp. [108]. They were identified as the mirror images of plant-derived caryolanes [109]. This discovery may point to complex cross-talk between plant and endophytic microorganisms [20].

### 4.3. Diterpenoids

**Figure 14:**
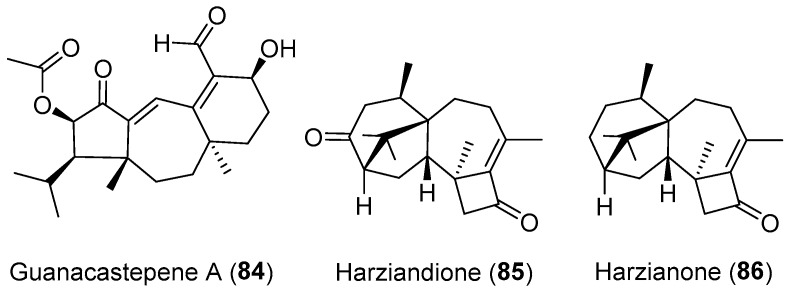
Structures of diterpenoids **84**–**86**.

From an unidentified fungus colonizing the plant *Daphnopsis americana*, guanacastepene A (**84**, Figure 14) and 14 biosynthetically related congers that comprised a unique family of diterpene natural products were found [110,111]. Compound **84** showed potent antibacterial activity against MRSA and vancomycin-resistant *Enterococcus faecalis* (VREF) through disrupting the cell membrane with inhibition zones of 11 and 9 mm at 100 μg per paper disk, respectively [111,112]. It has attracted numerous synthetic efforts or strategies toward the guanacastepenes [113,114]. Harziandione (**85**, Figure 14) and harzianone (**86**, Figure 14) are antimicrobial harziane diterpenes containing a unique tetracyclic scaffold from the potential biocontrol agents, *Trichoderma* spp. [115].

### 4.4. Sesterterpenoids

**Figure 15:**
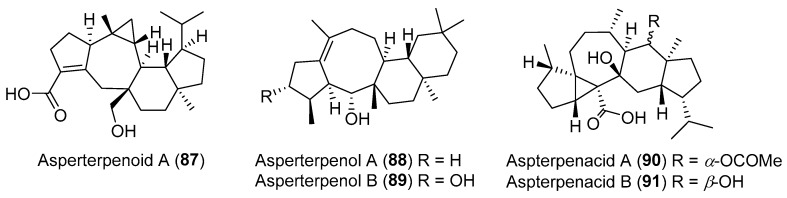
Structures of sesterterpenoids **87**–**91**.

The group of She has been dedicated to the search for structurally unique and biologically active compounds from mangrove plant-derived fungal endophytes, especially *Aspergillus* spp. Five sesterterpenoids with an unprecedented carbon skeleton including asperterpenoid A (**87**), asperterpenols A and B (**88** and **89**), and aspterpenacids A and B (**90** and **91**), have been obtained (Figure 15) [116,117,118]. Among them, compound **87** with an unprecedented 5/7/(3)6/5 pentacyclic system, inhibited the *Mycobacterium tuberculosis* protein tyrosine phosphatase B with an IC_50_ value of 2.2 μM [116]. Compounds **88** and **89** possessing an unusual 5/8/6/6 tetracyclic ring skeleton, exhibited inhibitory activity against acetylcholinesterase (AChE) with IC_50_ values of 2.3 μM and 3.0 μM, respectively [118]. There were no antibacterial and cytotoxic activities for compounds **90** and **91** with a rare carbon skeleton of a 5/3/7/6/5 ring system [117].

## 5. Hybrid Products

### 5.1. PKS-NRPS

**Figure 16:**
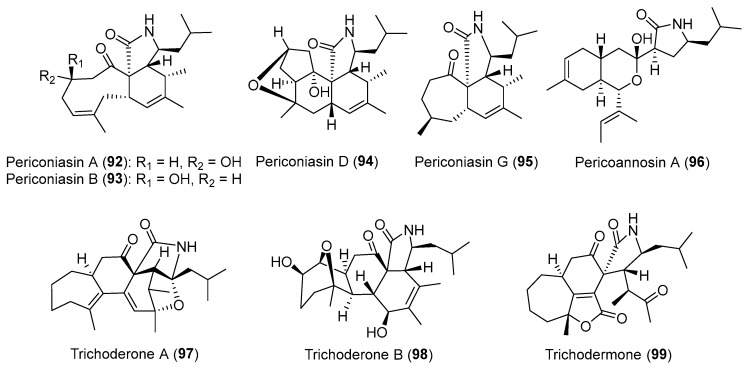
Structures of compounds **92**–**99**.

Cytochalasans are a large class of fungal secondary metabolites with biological diversity originating from a mixed PKS and nonribosomal peptide synthetase (NRPS) [119]. The group of Dai isolated an endophytic fungus *Periconia* sp. from the medicinal plant *Annona muricata*, and discovered it was cytotoxic to several human cancer cell lines. Bioassay-guided isolation of EtOAc extracts of the different fermentation media of this strain resulted in the isolation and identification of twelve novel PKS-NRPS hybrid cytochalasans [120,121,122,123,124]. Among them, periconiasins A and B (**92** and **93**, Figure 16) with an unprecedented 9/6/5 tricyclic ring system exhibited significant cytotoxicity against human HCT-8 cancer cells with IC_50_ values of 0.9 and 0.8 μM, respectively [123]. Periconiasin D (**94**, Figure 16) has a 5/6/6/5 tetracyclic ring skeleton, while periconiasin G (**95**, Figure 16) is the first cytotoxic cytochalasan with a 7/6/5 tricyclic ring system [120]. Pericoannosin A (**96**, Figure 16) possesses an unusual hexahydro-1*H*-isochromen-5-isobutylpyrrolidin-2-one skeleton and showed moderate anti-HIV activity (IC_50_ of 69.6 μM) [122,124]. Compounds **92**–**96** were proposed to be biosynthesized from an unusual seven acetate/malonate polyketide chain attached to a leucine unit by a PKS-NRPS and a key Diels-Alder reaction should be occurred in the cyclization of cytochalasans [122,123]. Owing to their structural diversity and biological activities, they have emerged as targets for bioinspired total syntheses [125].

**Figure 17:**
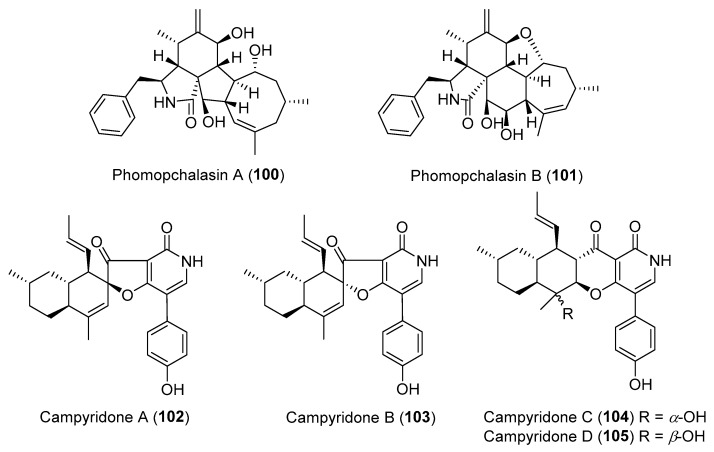
Structures of phomopchalasins A–B (**100**–**101**) and campyridones A–D (**102**–**105**).

From a fungal endophyte *Trichoderma gamsii* isolated from the traditional Chinese herb *Panax notoginseng*, three more unique cytochalasans, trichoderones A (**97**) and B (**98**) (Figure 16) together with trichodermone (**99**, Figure 16), were obtained by Zou and co-workers [126,127]. Their structures with an unprecedented pentacyclic or tetracyclic ring system might originate from a key intramolecular Michael 1,4-addition of the possible biosynthetic precursor aspochalasin D [126,127]. Compounds **97** and **98** showed weak inhibitory activity against the HeLa cell lines with IC_50_ values over 40 μM [127]. Recently, phomopchalasins A (**100**) and B (**101**) (Figure 17), two novel cytochalasans featuring unprecedented 5/6/5/8-fused tetracyclic or 5/6/6/7/5-fused pentacyclic skeletons, were isolated from the endophytic fungus *Phomopsis* sp., and compound **101** showed antimigratory activity against MDA-MB-231 with IC_50_ value of 19.1 μM [128].

Chemical investigation of a mangrove-derived endophytic fungus *Campylocarpon* sp., led to the isolation of four novel cytotoxic 4-hydroxy-2-pyridone alkaloids, campyridones A–D (**102**–**105**, Figure 17) [129]. Their unprecedented ring systems containing a spiro-furanone or *γ*-pyrone substructure were proposed to be synthesized by the PKS-NRPS hybrid involving a polyketide chain and a tyrosine moiety.

**Figure 18:**
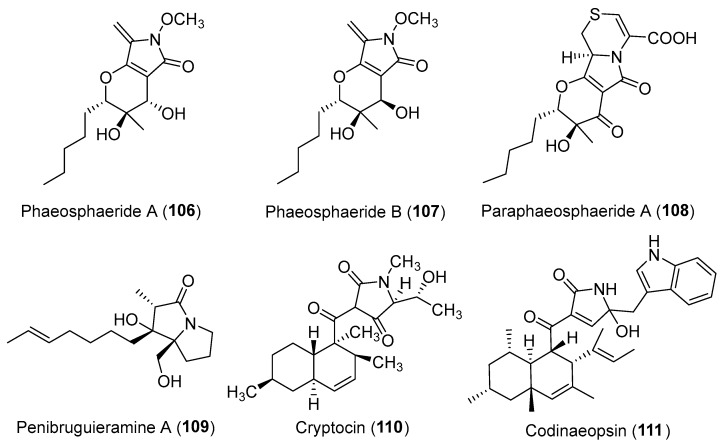
Structures of compounds **106**–**111**.

**Scheme 6:**
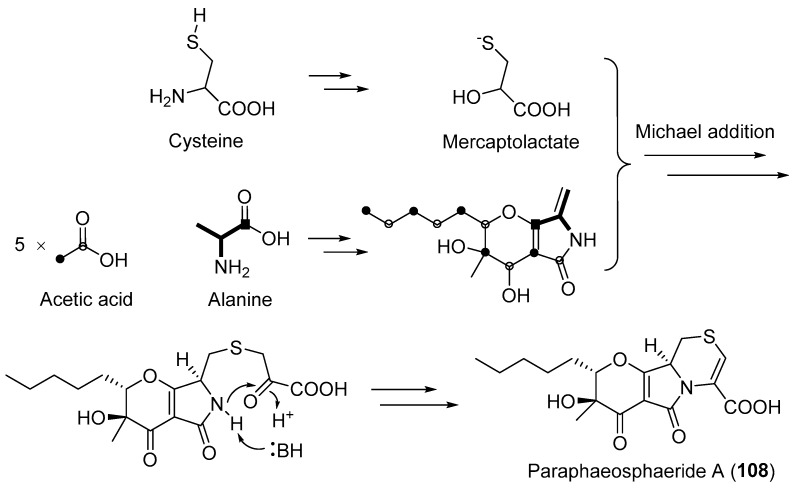
Proposed biosynthetic pathway for paraphaeosphaeride A (**108**).

Clardy and co-workers reported the isolation of two hybrid PKS-NRPS products: phaeosphaeride A (**106**, Figure 18) and its inactive diastereomer phaeosphaeride B (**107**, Figure 18) from an endophytic fungus *Phaeosphaeria avenaria* [130]. They were potent inhibitors of signal transducer and activator of transcription 3 (STAT3) signaling with an IC_50_ of 0.61 mM [130]. Their structural elucidations were achieved by spectral data [130], total synthesis [131,132] and X-ray crystallographic analysis [133]. The diastereomers or semi-synthetic derivatives of compounds **106** and **107** exhibited in vitro cytotoxicity against MD-MB-231, PANC-1, and A549 cancer cell lines [134,135]. Another biosynthetically related hybrid PKS-NRPS product, paraphaeosphaeride A (**108**, Figure 18), was discovered from an endophytic *Paraphaeosphaeria neglecta* isolated from the stem of Hawaiian-plant *Lycopodiella cernua* [136]. It has an unusual 4-pyranone-*γ*-lactam-1,4-thiazine moiety and showed STAT3 inhibition at 10 μM. The plausible hybrid biosynthetic pathway of compound **108** involving a precursor cysteine has been shown in Scheme 6 [136,137].

A proline-pentaketide amide, penibruguieramine A (**109**, Figure 18) with an unprecedented 1-hydroxy-2-methylpyrrolizidin-3-one skeleton, was isolated from *Penicillium* sp. associated with the Chinese mangrove *Bruguiera gymnorrhiza* [138]. A biomimetic total synthesis of compound **109** involving a key intramolecular aldol-type reaction was accomplished by Kim et al. [139]. The endophytic fungus *Cryptosporiopsis cf. quercina* produced a unique functionalized tetramic acid, cryptocin (**110**) (Figure 18) arising from a mixed PKS-NRPS pathway [140,141]. It demonstrated significant inhibitory activity against a wide variety of plant pathogens, including the fungus *Pyricularia oryzae* (the causal agent of rice blast disease) with an MIC value of 0.39 μg/mL [140]. Further total synthesis, semi-synthetic and biological studies by the group of Gao suggested the importance of different tetramic acid ring systems for cytotoxicity [142]. A high-throughput screen for endophytes-derived antimalarial compounds enabled the discovery of a new tryptophan-polyketide hybrid with a polyketide decalin [141], codinaeopsin (**111**, Figure 18) [143]. Compound **111** had an IC_50_ of 4.66 μM against *P. falciparum*, the causative agent of the most lethal form of malaria.

### 5.2. NRPS-Terpene

**Figure 19:**
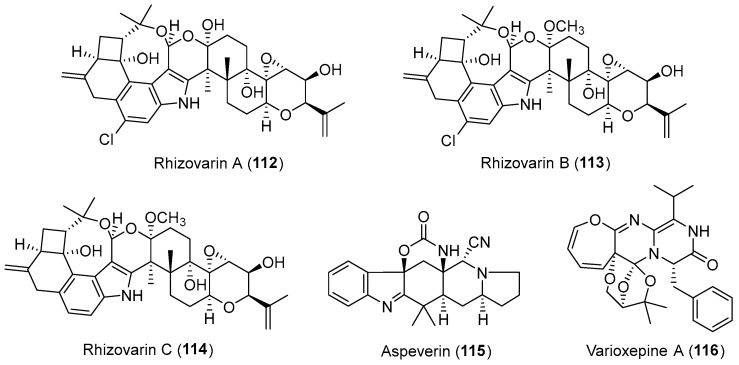
Structures of compounds **112**–**116**.

A mangrove-derived endophyte *Mucor irregularis* was found to produce three novel indole-diterpenes [144], named rhizovarins A–C (**112**–**114**, Figure 19) [145]. They appeared to be chemically unique due to the complex 4,6,6,8,5,6,6,6,6-fused indole-diterpene ring system that incorporates an unusual acetal linked to a hemiketal (**112**) or a ketal (**113** and **114**). Compounds **112** and **113** were effective against the human A-549 (IC_50_: 11.5 μM for **112**; 6.3 μM for **113**) and HL-60 cancer cell lines (IC_50_: 9.6 μM for **112**; 5.0 μM for **113**) [145].

The group of Ji isolated and identified a novel prenylated indole alkaloid, aspeverin (**115**, Figure 19) from an endophytic strain *Aspergillus versicolor* harbored in the marine green alga *Codium fragile* [146]. It showed inhibitory activity against marine phytoplankton (*Heterosigma akashiwo*) with the EC_50_ values of 16.7 and 9.0 μM for 24 and 96 h, respectively. The structure of compound **115** containing an unprecedented cyclic carbamate linkage and a rare cyano could be assembled through a dipeptide-like precursor with dimethylallyl pyrophosphate (DMAPP) [147,148], and has promoted the attention of chemists from a totally synthetic perspective [149]. Varioxepine A (**116**, Figure 19), a 3*H*-oxepine-containing alkaloid with an unprecedented oxa-cage unit, was isolated from *Paecilomyces variotii*, an endophytic fungus residing in marine red alga [150]. It showed diverse antibacterial activities with MIC values ranging from 16 to 64 μg/mL and inhibited plant pathogenic fungus *Fusarium graminearum* with an MIC value of 4 μg/mL. Like compounds **59**–**60**, compound **116** could be biosynthesized by the condensation of ATA, valine, phenylalanine, and DMAPP [151].

### 5.3. PKS-Terpene

**Figure 20:**
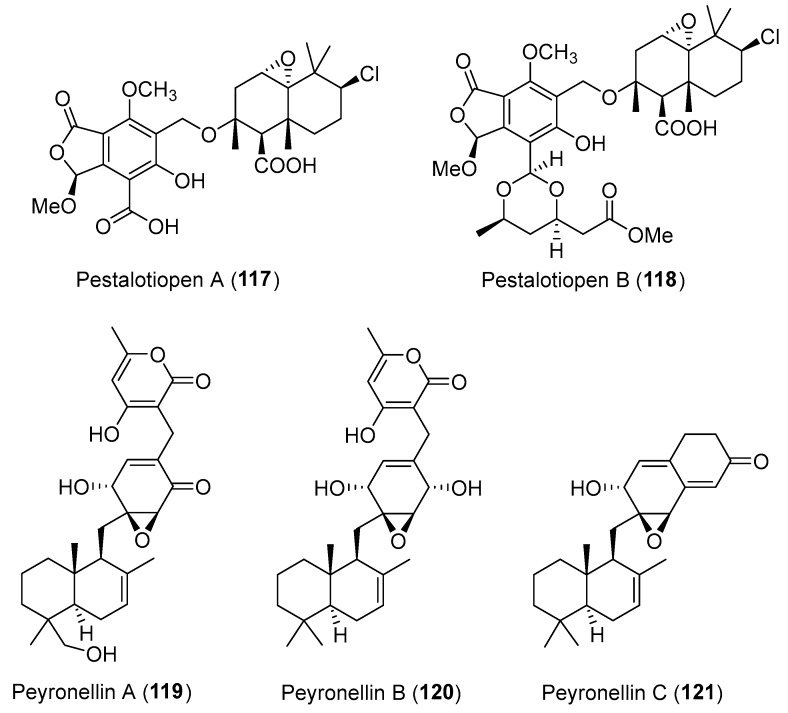
Structures of pestalotiopens A–B (**117**–**118**) and peyronellins A–C (**119**–**121**).

Two novel hybrid sesquiterpene-cyclopaldic acid metabolites, named pestalotiopens A (**117**) and B (**118**) (Figure 20), were obtained from the marine endophytic fungus *Pestalotiopsis* sp. [152]. Compound **117** showed moderate antibacterial activity against *E. faecalis* whereas compound **118** containing a third, triketide-derived moiety was inactive. Three unusual polyketide-sesquiterpene metabolites peyronellins A–C (**119**–**121**, Figure 20), have been isolated from the endophytic fungus *Peyronellaea coffeae-arabicae*, which was isolated from the native Hawaiian plant *Pritchardia lowreyana* [153]. Compound **119** was active against A2780 and A2780 CisR cancer cell lines with IC_50_ values of 1.8 and 3.4 μM, respectively, while compounds **120** and **121** were inactive.

### 5.4. PKS-NRPS-Terpene

**Figure 21:**
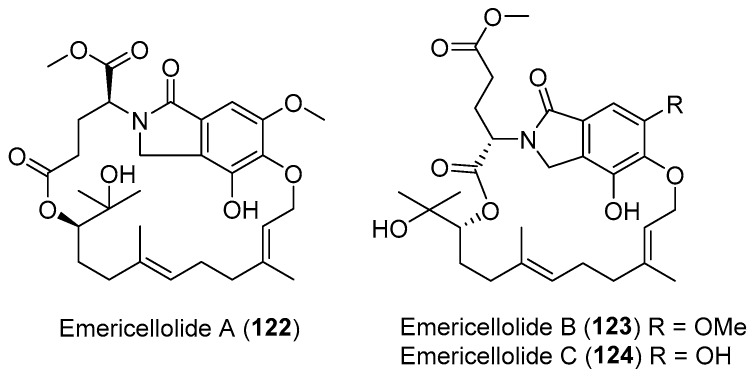
Structures of emericellolides A–C (**122**–**124**).

From a plant endophytic fungus *Emericella nidulans*, emericellolides A–C (**122**–**124**, Figure 21) with an unprecedented macrolide skeleton were found by Li and co-workers. A l-glutamate fragment, an isoindolone unit [154], and a sesquiterpene moiety might be involved in the construction of the macro-ring in compounds **122**–**124** [155].

**Figure 22:**
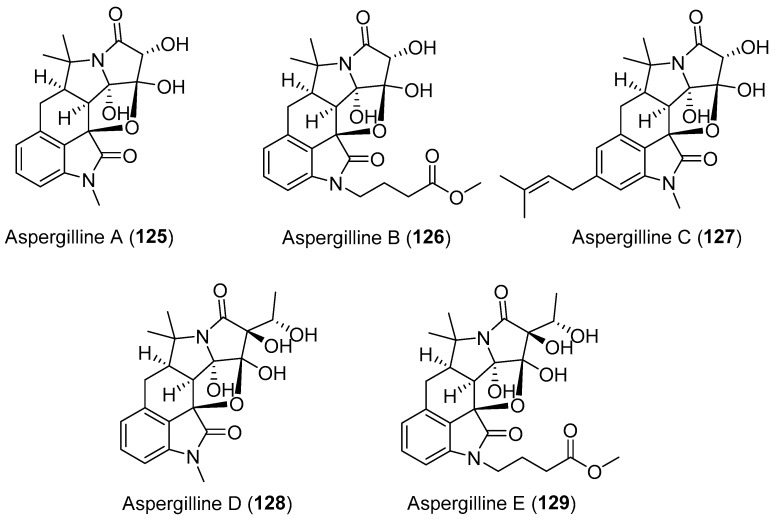
Structures of aspergillines A–E (**125**–**129**).

An endophytic fungus *Aspergillus versicolor* was isolated from the rhizome of *Paris polyphylla* var. *yunnanensis* by Zhou et al. and was found to biosynthesize five highly oxygenated cyclopiazonic acid-derived alkaloids, aspergillines A–E (**125**–**129**, Figure 22) [156,157]. Compounds **125**–**129** with a rigid hexacyclic (6/5/6/5/5/5) indole-tetrahydro-furan-tetramic acid scaffold, were proposed to arise from a mixed biosynthetic pathway that involves a tryptophan unit, one or two molecules of acetate, and DMAPP [158,159,160]. They not only exhibited significant anti-tobacco mosaic virus (anti-TMV) activity with IC_50_ values of 15.4–48.6 μΜ, but also showed moderate cytotoxicity against a panel of human cancer cell lines [157].

## 6. Conclusions

**Figure 23:**
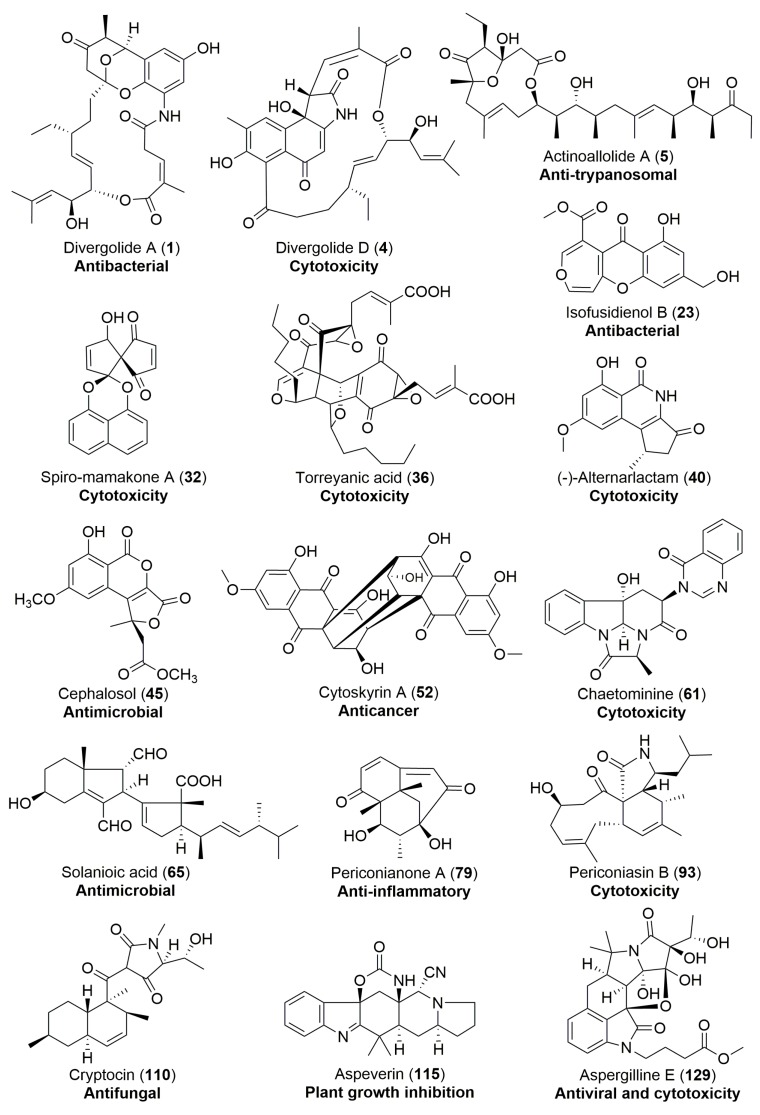
Selected endophyte-derived secondary metabolites with significant biological activities.

Stereochemically complex and structurally diverse secondary metabolites play a pivotal role in discovery campaigns for new natural product drug pharmacophores. A number of structurally novel compounds are increasingly being discovered from endophytic fungi and bacteria and could comprise a powerful compound library for drug lead development. Herein, we present a comprehensive review of 129 endophyte-derived natural products with new carbon skeletons, unusual ring systems, or rare structural moieties (Table 1). Most of them were discovered from fungal endophytes in which more than 70% were isolated from terrestrial plants, especially those with an ethnobotanical history. The structural novelty and diversity of these microbial metabolites are as a result of the enormous diversity of terrestrial and marine endophytes in combination with their potential biosynthetic capabilities. In addition, they display diverse and remarkable biological activities, and frequently reported biological properties are antimicrobial and cytotoxic activities (Table 1). As shown in Figure 23, 16 secondary metabolites with marked biological activities might deserve more attention from chemists and biologists in further investigations.

Although traditional bioassay-guided chemical investigation encounters the frequent re-isolation of known compounds, it remains the most popular approach in discovering structurally novel small molecules from endophytes, especially with the aid of advanced analytical techniques, such as LC-MS. Alteration of easily accessible cultivation parameters, such as media composition, has well proven in this review to activate the silent gene clusters in endophytes and will continue to be used as a promising strategy for increasing the number of novel natural products by a single microbial strain.

Furthermore, recent advances in microbial genomics and metagenomics offer promising opportunities to access cryptic secondary metabolites. It is expected that most endophytic species are more likely to be uncultivable or poorly cultivable in standard laboratory conditions. Exploration of this largely unexplored source would provide more structurally unique compounds with properties suitable for a wide variety of biological and medicinal applications.

## Figures and Tables

**Table 1:** Structurally novel natural products from endophytic bacteria or fungi.

Microorganisms	Origin	Secondary Metabolites (Figures)	Reported Activities *^a^*	Ref.
*Streptomyces *sp.	Marine, *Bruguiera gymnorrhiza*	Divergolides A–D (**1**–**4**) (Figure 1)	Antibacterial, cytotoxicity	[28]
*Streptomyces* sp.	Terrestrial, *Camellia sinensis*	Rubrolone B (**43**) (Figure 7)	NR *^b^*	[61]
*Streptomyces* sp.	Marine, *Bruguiera gymnorrhiza*	Bacaryolanes A–C (**81**–**83**) (Figure 13)	NR	[108]
*Chalara* sp.	Terrestrial, *Artemisia vulgaris*	Isofusidienols A–D (**22**–**25**) (Figure 4)	Antibacterial	[39]
*A nonsporulating fungus*	Terrestrial, *Knightia excelsa*	Spiro-mamakone A (**32**) (Figure 5)	Antibacterial, cytotoxicity	[45]
*Penicillium brocae*	Marine	Spirobrocazines A–B (**57**–**58**) (Figure 10)	Antibacterial, cytotoxicity	[83]
*Pestalotiopsis fici*	Terrestrial	Chloropupukeananin (**72**) (Figure 13)	Antibacterial, antiviral	[100]
Unidentified fungus	Terrestrial, *Daphnopsis americana*	Guanacastepene A (**84**) (Figure 14)	Antibacterial	[110,111]
*Blennoria* sp.	Terrestrial, *Carpobrotus edulis*	Blennolides A–G (**11**–**17**) (Figure 3)	Antifungal, antialgal	[36]
*Cryptosporiopsis* sp.	Terrestrial, *Viburnum tinus*	Viburspiran (**48**) (Figure 8)	Antifungal	[74]
*Cryptosporiopsis cf. quercina*	Terrestrial, *Triptergyium wilfordii*	Cryptocin (**110**) (Figure 18)	Antifungal	[140]
*Cephalosporium acremonium*	Terrestrial, *Trachelospermum jasminoides*	Cephalosol (**45**) (Figure 8)	Antimicrobial	[68]
*Daldinia eschscholtzii*	Terrestrial, *Paphiopedilum exul*	Daldionin (**47**) (Figure 8)	Antimicrobial	[73]
*Rhizoctonia solani*	Terrestrial, *Cyperus rotundus*	Solanioic acid (**65**) (Figure 12)	Antimicrobial	[94]
*Trichoderma* spp.	Marine, alga *Codium fragile*	Harziandione (**85**) and harzianone (**86**) (Figure 14)	Antimicrobial	[115]
*Paecilomyces variotii*	Marine	Varioxepine A (**116**) (Figure 19)	Antimicrobial	[150]
*Pestalotiopsis* sp.	Marine, *Rhizophora mucronata*	Pestalotiopens A–B (**117**–**118**) (Figure 20)	Antimicrobial	[152]
*Pestalotiopsis fici*	Terrestrial	Chloropestolide A (**73**) (Figure 13)	Anti-HIV, cytotoxicity	[102]
*Periconia* sp.	Terrestrial, *Annona muricata*	Pericoannosin A (**96**) (Figure 16)	Anti-HIV	[122]
*Neosartorya udagawae*	Marine, *Aricennia marina*	Neosartoryadins A–B (**59**–**60**) (Figure 11)	Antiviral	[84]
*Periconia* sp.	Terrestrial, *Annona muricata*	periconiasins (**92**–**95**) (Figure 16)	Antiviral, cytotoxicity	[120,121,123]
*Aspergillus versicolor*	Terrestrial, *polyphylla* var. *yunnanensis*	Aspergillines A–E (**125**–**129**) (Figure 22)	Antiviral, cytotoxicity	[157]
*Periconia* sp.	Terrestrial, *Annonsa muricata*	Periconianone A (**79**) (Figure 13)	Anti-inflammatory	[105]
Unidentified fungus	Terrestrial, *Vochysia guatemalensis*	Codinaeopsin (**111**) (Figure 18)	Antimalarial	[143]
*Phomopsis* sp.	Terrestrial, *Isodon eriocalyx var. laxiflora*	Phomopchalasins A–B (**100**–**101**) (Figure 17)	Antimigratory activity	[128]
*Fusarium pallidoroseum*	Terrestrial	Apicidins A–C (**62**–**64**) (Figure 11)	Antiprotozoal, anticancer	[90]
*Actinoallomurus fulvus*	Terrestrial, *Capsicum frutescens*	Actinoallolides A–E (**5**–**9**) (Figure 2)	Anti-trypanosomal	[34]
*Aspergillus *sp*.*	Marine	Asperterpenoid A (**87**) (Figure 15)	Antituberculosis	[116]
*Aspergillus *sp*.*	Marine	Asperterpenols A–B (**88**–**89**) (Figure 15)	Acetylcholinesterase inhibition	[118]
*Cytospora* sp.	Terrestrial, *Conocarpus erecta*	Cytoskyrins A–B (**52**–**53**) (Figure 9)	BIA activity	[78]
Unidentified fungus	Terrestrial, *Ficus microcarpa *L.	Microcarpalide (**10**) (Figure 2)	Cytotoxicity	[35]
*Nodulisporium* sp.	Marine, Alga	Noduliprevenone (**18**) (Figure 3)	Cytotoxicity	[37]
*Pestalotiopsis virgatula*	Terrestrial, *Dracontomelon duperreanum *	Virgatolides A–C (**27**–**29**) (Figure 5)	Cytotoxicity	[41]
*Pestalotiopsis microspora*	Terrestrial, *Torreya taxifolia*	(±)-torreyanic acid (**36**) (Figure 6)	Cytotoxicity	[50]
*Chaetomium globosum*	Terrestrial, *Imperata cylindrical*	Chaetoglobins A–B (**38**–**39**) (Figure 7)	Cytotoxicity	[56]
*Alternaria* sp.	Terrestrial, *Carex aridula*	(-)-Alternarlactam (**40**) (Figure 7)	Cytotoxicity	[57]
*Penicillium manginii*	Terrestrial, *Panax notoginseng*	Duclauxamide A1 (**42**) (Figure 7)	Cytotoxicity	[59]
*Penicillium* sp.	Terrestrial, *Catharanthus roseus*	Citreoviripyrone A (**46**) (Figure 8)	Cytotoxicity	[71]
*Chaetomium* sp.	Terrestrial, *Adenophora axilliflora*	Chaetominine (**61**) (Figure 11)	Cytotoxicity	[86]
*Pestalotiopsis fici*	Terrestrial	Chloropupukeanolides A–E (**74**–**78**) (Figure 13)	Cytotoxicity	[103,104]
*Trichoderma gamsii*	Terrestrial, *Panax notoginseng*	Trichoderones A–B (**97**–**98**) (Figure 16)	Cytotoxicity	[127]
*Trichoderma gamsii*	Terrestrial, *Panax notoginseng*	Trichodermone (**99**) (Figure 16)	Cytotoxicity	[126]
*Campylocarpon* sp.	Marine, *Sonneratia caseolaris*	Campyridones A–D (**102**–**105**) (Figure 17)	Cytotoxicity	[129]
*Mucor irregularis*	Marine, *Rhizophora stylosa*	Rhizovarins A–C (**112**–**114**) (Figure 19)	Cytotoxicity	[145]
*Peyronellaea coffeae-arabicae*	Terrestrial, *Pritchardia lowreyana*	Peyronellins A–C (**119**–**121**) (Figure 20)	Cytotoxicity	[153]
*Pestalotiopsis sp.*	Terrestrial, *Taxus brevifolia*	Pestalotiopsin A (**80**) (Figure 13)	Immunosuppressive	[107]
*Penicillium dangeardii*	Terrestrial, *Lysidice rhodostegia*	Penicillactones A–C (**33**–**35**) (Figure 5)	Inhibitors of the release of *β*-glucuronidase	[48]
*Phaeosphaeria avenaria*	Terrestrial	Phaeosphaeride A–B (**106**–**107**) (Figure 18)	Inhibiting STAT3 activity	[130]
*Aspergillus versicolor*	Marine, green alga *Codium fragile*	Aspeverin (**115**) (Figure 19)	Marine plant growth inhibition	[146]
*Microsphaeropsis* sp.	Terrestrial, *Lycium intricatum*	Microsphaeropsones A–C (**19**–**21**) (Figure 4)	NR	[38]
*Paraphaeosphaeria neglecta*	Terrestrial, *Lycopodiella cernua*	Lycopodiellactone (**26**) (Figure 4)	NR	[40]
*Pestalotiopsis virgatula*	Terrestrial, *Terminalia chebula*	Pestalospiranes A–B (**30**–**31**) (Figure 5)	NR	[43]
*Pestalotiopsis* sp.	Terrestrial, *Clavaroids *sp.	Torreyanic acid analogue (**37**) (Figure 6)	NR	[53]
*Fusarium* sp.	Terrestrial, *Melia azedarach*	Fusarimine (**41**) (Figure 7)	NR	[58]
*Fusidium sp.*	Terrestrial, *Mentha arvensis*	Fusidilactone C (**44**) (Figure 8)	NR	[65,66]
*Corynespora cassiicola*	Marine, *Laguncularia racemosa*	Coryoctalactone E (**49**) (Figure 8)	NR	[75]
*Colletotrichum capsici*	Terrestrial, *Siegesbeckia pubescens*	Citrinals A–B (**50**–**51**) (Figure 9)	NR	[76,77]
*Aspergillus* sp.	Terrestrial, *Melia azedarach* L.	Aspertryptanthrins A–C (**54**–**56**) (Figure 10)	NR	[82]
*Chalara alabamensis*	Terrestrial, *Asterogyne martiana*	Asterogynins A–B (**66**–**67**) (Figure 12)	NR	[96]
*Talaromyces wortmannii*	Terrestrial, *Tripterygium wilfordii*	Wortmannines A–C (**68**–**70**) (Figure 12)	NR	[98]
*Talaromyces wortmanni*	Terrestrial, *Tripterygium wilfordii*	Secovironolide (**71**) (Figure 12)	NR	[97]
*Aspergillus *sp*.*	Marine, *Kandelia obovata*	Aspterpenacids A–B (**90**–**91**) (Figure 15)	NR	[117]
*Paraphaeosphaeria neglecta*	Terrestrial, *Lycopodiella cernua*	Paraphaeosphaeride A (**108**) (Figure 18)	NR	[136]
*Penicillium* sp.	Marine, *Bruguiera gymnorrhiza*	Penibruguieramine A (**109**) (Figure 18)	NR	[138]
*Emericella nidulans*	Terrestrial, *Tamarix chinensis* Lour	Emericellolides A–C (**122**–**124**) (Figure 21)	NR	[155]

*^a^* Organize the bioactivity using alphabetical order. ^*b*^ NR: not reported in references or have reported in literature to have no biological activities.
